# Extension of Dancer’s Legs: Increasing Angles Show Motion

**DOI:** 10.3389/fpsyg.2021.706004

**Published:** 2022-01-04

**Authors:** Stefano Mastandrea, John M. Kennedy

**Affiliations:** ^1^Department of Education, Roma Tre University, Rome, Italy; ^2^Department of Psychology, University of Toronto, Toronto, ON, Canada

**Keywords:** motion, dancer, leg, extension, difficulty, liking, static pictures

## Abstract

Usain Bolt’s *Lightning Bolt* pose, one arm highly extended to one side, suggests action. Likewise, static pictures of animals, legs extended, show animation. We tested a new cue for motion perception—extension—and in particular extension of dancer’s legs. An experiment with pictures of a dancer finds larger angles between the legs suggest greater movement, especially with in-air poses and in lateral views. Leg positions graded from simply standing to very difficult front and side splits. *Liking* ratings (a small range) were more related to *Difficulty* ratings (a large range) than *Movement* ratings (a moderate range).

## Introduction

Though it is static, Usain Bolt’s *Lightning Bolt* pose, one arm highly extended to one side, suggests vigorous action. Likewise, in pictures of people and animals, leg spread may suggest animation.

Greatly admired poses in profile and frontal view suggest motion. For example, in a frontal view, Leonardo’s “Vetruvian Man” elevates his arms and separates his legs. Further, in a side view, Gericault’s *Derby* horses are in a flying gallop, legs stretched out fore and aft. Indeed, the further a horse spreads its legs, the speedier it seems, but observers deem the flying gallop unrealistic (Mastandrea and Kennedy, 2018). Comic books use motion lines and blur to suggest action (Gombrich, 1964; Kennedy, 1982; Carello et al., 1986; Cohn, 2013). The devices are often omitted if pose biomechanics are obvious (Juricevic, 2017). The pictorial devices activate motion-sensitive cortex (Kourtzi and Kanwisher, 2000; Cattaneo et al., 2015), and in a study on observers examining photographs of human actions, implied motion increased cortical activation (Proverbio et al., 2009). Cortical activation is particularly strong for pictured movements rated as *pleasing* and *difficult* to reproduce (Cross et al., 2011). Cortical activation levels might indicate amount of depicted motion perceivers report, and impressions of the perceived position of stationary objects (Pinna and Brelstaff, 2000; Pavan et al., 2011). A cortical AON or *action observation network* is thought to be triggered by static images and might be related to the observer’s own skills (Orlandi et al., 2020b).

Research into dance aesthetics has often examined subjective features such as familiarity, Orlandi et al. (2020a) note, pointing out that the studies rarely studied objective features. Research on observation of dance styles varies aggregations of features (Calvo-Merino et al., 2005). Similarly, research on observing motion and dance structure considers *combinations* of poses, *good continuation* and the *grammar* of dance (Orgs et al., 2013). Here we focus on one feature—extension of legs with increasing angles between the legs.

In art, wide limb spread has been used to convey extremes of movement. A celebrated *Scots Greys Charging* oil painting from 1881 by Lady Butler shows horses from *in front* in the center of the picture, and to the side of the image shows horses in *lateral view*. Most of the hooves in the picture are off the ground, front legs reaching forward, and rear legs backward. In contrast, a lateral picture of a rearing horse, back legs both on the ground, almost vertical, a very difficult pose to maintain, Jacques-Louis David’s *Napoleon Crossing the Alps*, from 1801, suggests motion upwards has occurred. Mastandrea and Kennedy (2018) sampled pictures of horses from the art world and found liking was correlated positively with motion. However, they included unrealistic limb poses and, since the stimuli were horses, they were not concerned with ratings of difficulty for human participants.

Here, we test photographs of a dancer varying in limb spread—the angle between extended legs. Will motion ratings increase with spread? In addition to the Cutting (2002) indicators of movement in pictures (broken symmetry, multiple images, forward lean, blur, action lines), leg extension, varying angles between legs, may suggest amount of motion. Of interest, Arnheim (1974) suggested liking would increase with the display’s dynamic qualities. Cross et al. (2011) suggest the skill level or difficulty of a pose may be more influential. Various relationships between movement, difficulty and liking have been reported. The typical viewer encodes effortful movements in a less refined way than professional dancers (Orlandi et al., 2020b). Does this mean ordinary observers do not find movement, difficulty and liking highly related? In observations of professional dancers undertaking complex dance movements, Cross et al. (2011) found a positive relation between difficulty and liking. Also, a positive relationship between amount of practice of small-muscle (eye muscle) motions and liking of observed images was reported by Topolinski (2010). At an extreme, difficulty might appear unpleasant. Much may depend on the poses considered and their range. Here we consider a dancer in a range of formal poses covering from simply standing to the full splits.

If a picture is from an unrevealing vantage point, being literally correct is no assurance of information for dynamics and biomechanics (Heller et al., 2002; Mastandrea and Umiltà, 2016). In this study we investigate if leg angles—limb spread—have implications for motion. As the angle between the legs increases, is more motion indicated? We test this hypothesis with photographs of a dancer in different realistic poses. We used pictures of a dancer in-air, capturing a frozen moment of vigorous action, and ones of a dancer on the ground, in which motion might be implied as in *Lightning Bolt.* Leg spread was symmetrical about vertical, and we provided views in which both legs and their spread was always obvious, not obscured. Under these conditions, Gibson (1979) and Di Dio et al. (2016) argued that sensory arrays provide rich information for dynamics. Leg angle matters for ground-based terrestrial animals such as horses and people—quadrupeds and bipeds—offering support at rest when vertical and on-ground, and suggesting motion and effort at other angles and in-air.

**FIGURE 1:**
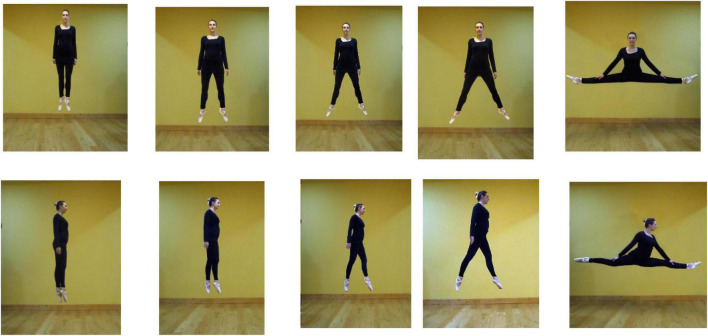
Frontal (top) and lateral (bottom) poses of the dancer in air.

**FIGURE 2:**
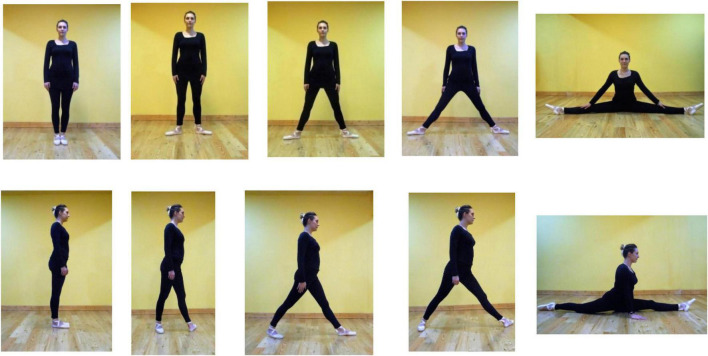
Frontal (top) and lateral (bottom) on-ground poses of the dancer.

We asked participants to rate Figures 1, 2 for motion, expecting increases with spread. We also asked for likability and difficulty ratings. Since the photographs are of a professional dancer in formal poses, we expected all the pictures to be likeable. The range of likeability ratings might be modest as a result. Also, since the dancer was shown in poses from standing on the ground to the splits in the air and on-ground, and few ordinary observers can undertake the splits, the range of difficulty ratings might be wide. Of interest, the likability scores could be more related to motion ratings or more to difficulty ratings (Arnheim, 1974; Orgs et al., 2013; Orlandi et al., 2020b).

Our participants viewed pictures of a dancer in poses viewed from the front and the side, *on-ground* and jumping up (*in-air*), legs varying from parallel to widely separated in the splits, fully extended to the dancer’s left and right, or fore and aft. The postures include transient and sustained ones. Some have support from the ground, some have none. In-air poses are *literally* captured in-motion. Those on-ground at best *imply* motion. The issues we consider are: Do motion ratings increase reliably across the conditions, as limb angle increases? Or only in literal conditions i.e., in-air? How do lateral views compare to frontal views? Also, for likeability, is amount of movement outranked by difficulty?

## Materials and Methods

### Participants

Following standard practice at the university, potential subjects were contacted on-line, through the student social group of the Roma Tre University’s Department of Education. After agreeing to participate, students were invited to the Psychology Laboratory at the Department. A total of 100 participants volunteered (61 women, 39 men; mean age 35.2, SD 13.6). They reported no expertise in art and no professional dance experience. They had normal or corrected-to-normal vision and were naive about the purpose of the experiment. To participate in the study, they signed a written consent form.

### Stimuli

The stimuli were 20 high-quality colored digital photographs of a dancer, in 4 sets of 5 (Figures 1, 2), shown via a monitor. Picture sizes in the display were between 12 and 18 cm in height and between 10 and 14 cm in width, with display resolution 36 dots per cm. They showed a female professional dancer, hair tied together at the back of the head, in white ballerina soft shoes and neck-to-ankle form-fitting black clothes—a dance outfit, leotard and top—set against a dark greenish-yellow textured ground and a plain lighter-colored wall. The dancer was pictured facing front and facing to the right (a lateral view). Arms were down except in splits (arms 40° to the body in this case) since in on-ground splits poses the arms reach the ground.

The leg-spread extents in the images range from 0 to 180°, measuring from heel to heel, vertex at the crotch. The intermediate angles are variable. For simplicity, we will refer to the angles in order by their means, rounded to the nearest 5° e.g., 20^°^, 40^°^, and 65°.

Photographs show a *frozen moment* and can specify an action (Gibson, 1979). Dancer poses in air are brief and on-ground can be sustained. *A priori*, this can affect ratings of motion, difficulty and likeability.

Figure 1 shows the dancer in a jump—in-air—and from in-front (top row) and from the side i.e., lateral. In the leftmost images, the legs are parallel, the torso is erect, and the arms, straight, are by the side, elbows-in (*arms down* in ballet, the *first position* in the Cecchetti and Bournonville methods but with elbows-in). On the right, in splits, the legs are aligned horizontally.

In the top left image in Figure 1, the dancer’s legs are parallel, 0° apart, and the torso is in an erect posture. In the neighboring image the legs are 20° apart. In the middle image in the row the legs are 35° apart, and in the next image 55°. On the right, the legs are extended to the dancer’s left and right and aligned with each other. A skilled, difficult dance motion, it is a *side split*. We will refer to it as 180°.

In the lower row, the camera vantage point is from the dancer’s right side. The dancer’s head and body face right. After the leftmost picture—erect—one leg is to the fore and one to the rear. In the rightmost image, the legs are aligned horizontally, with a wide spread, 180°, fore-and-aft of the dancer. This is a *front split*, and difficult. In other poses the dancer jumps with her torso erect, feet pointed downwards. In order, the images in the bottom row are 0° spread (legs together), 10^°^, 25^°^, 45^°^, and 180°.

Figure 2 offers similar poses to Figure 1 but on the ground. The angles between the legs range from 0° (vertical legs) to 180° (horizontal).

In the top row, photographed from in front, on-ground, the legs spread to the left and right sides of the dancer. Lesser spreads (in order, left to right, 0°, 20°—roughly, second position in ballet—45^°^ and 80°) have feet flat on the ground. Two poses on the left (0° and 20°) are *standing* since the torso is vertically above the heels. This is biomechanically efficient. It is relatively static, though in practice, in maintaining upright stance, the body oscillates to and fro, mostly in the sagittal plane, like an inverted pendulum (Carello and Turvey, 2017). In 45^°^ and 80°spreads, the body is not vertically above the heels. The legs form acute angles with the ground, and are *straddling.* The heels are not below the torso, and compared to standing are inefficient biomechanically. In the extreme posture, the 180° side split, the ground supports the legs, but the pose is highly difficult.

The lower row of on-ground images in Figure 2 are taken from a vantage point on the right side of the dancer. The leftmost pose is standing, 0° leg separation. In the next three images from the left, the left foot is forward, as if the dancer was stepping. In the shortest step, leg separation 30°, the rear thigh is vertically aligned with the body, supporting the body against gravity, but both of the feet are wide of the torso, not under it. In the two longer strides (65^°^ and 80° leg separations), neither heel is under the torso. The 35^°^, 65^°^, and 80° leg-separation poses are biomechanically inefficient. The bottom-right image offers an especially difficult pose, a front split, legs close to alignment horizontally, 180° apart, the right knee on the ground, and the thigh raised. The contact with the ground in this pose can be difficult to sustain.

In Figure 1, top row, the frontal-view, the arms, are downwards in the sagittal plane of the body, and accommodate the leg spread, but in the splits the arms are about 40° to the body. To be comparable, in the splits in side-view each arm is again about 40° to vertical. In Discussion, we note that subtle variations in biomechanical efficiency, and arm and foot poses, are associated with limb spread in realistic photographs of dancers.

### Procedure

Participants were seated in an isolated laboratory room, in front of a 48 cm computer monitor located at a distance of about 60 cm from the participant’s head. The pictures were presented one at a time on the computer monitor. To randomize the presentation, we used four different power-point slide shows, with different orders of the pictures. Participants answered three questions, in Italian, here translated as (1) “How much movement is shown in the picture?” (2) “How much do you like this picture?” (3) “How difficult is it to assume this position?” The answers were given on a Likert scale, from 1 (not at all) to 5 (very much). Every participant was given a booklet; each page contained the three written questions. After the exposure to each image, participants were required to respond to the questions by checking a number on the scale, with no time limit. Once the assessment of one stimulus was completed he/she had to press the enter button to move to the next image and turn the booklet to the next page to answer the same three questions; this task was performed for all the 20 stimuli. The duration of the experiment was about 10 min.

## Results

Each participant responded to all the items and questions, giving 20 scores per scale. Offering details of the findings, mean scores (and SD) for each pose are in Tables 1–4.

**TABLE 1:** Frontal in-air poses: mean ratings (and SD) for movement, difficulty, and liking.

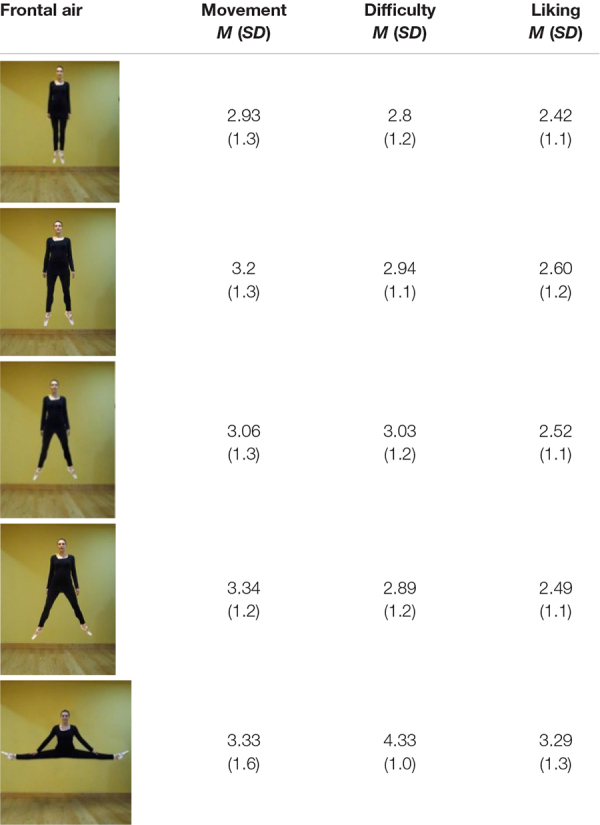

**TABLE 2:** Lateral in-air poses: mean ratings (and SD) for movement, difficulty, and liking.

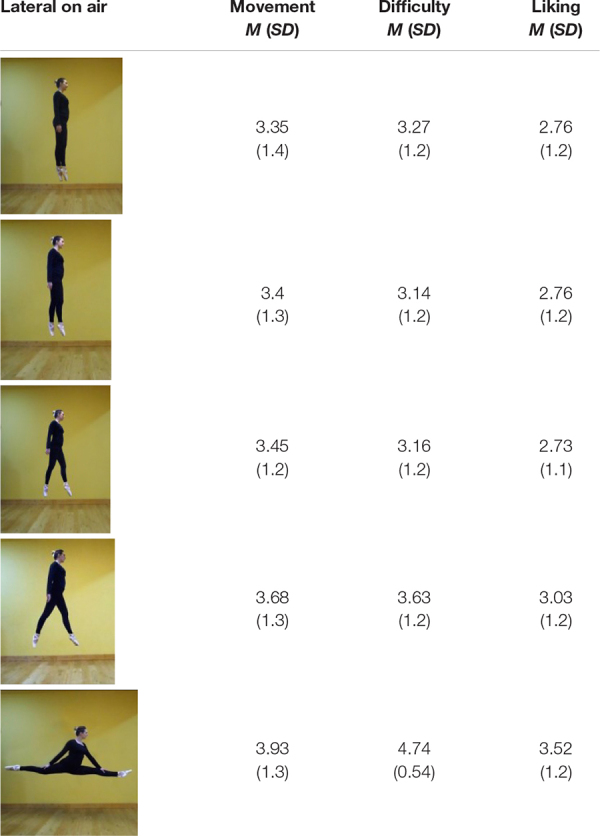

**TABLE 3:** Frontal on-ground poses: mean ratings (and SD) for movement, difficulty, and liking.

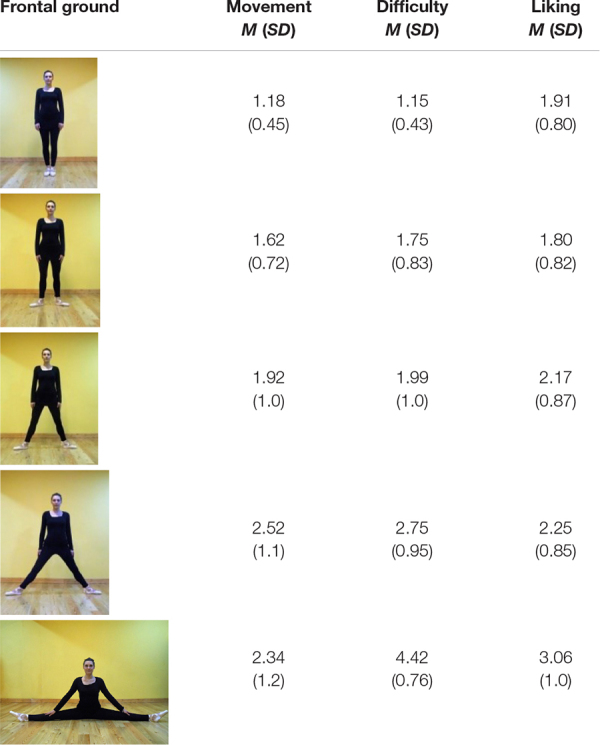

**TABLE 4:** Lateral on-ground poses: mean ratings (and SD) for movement, difficulty, and liking.

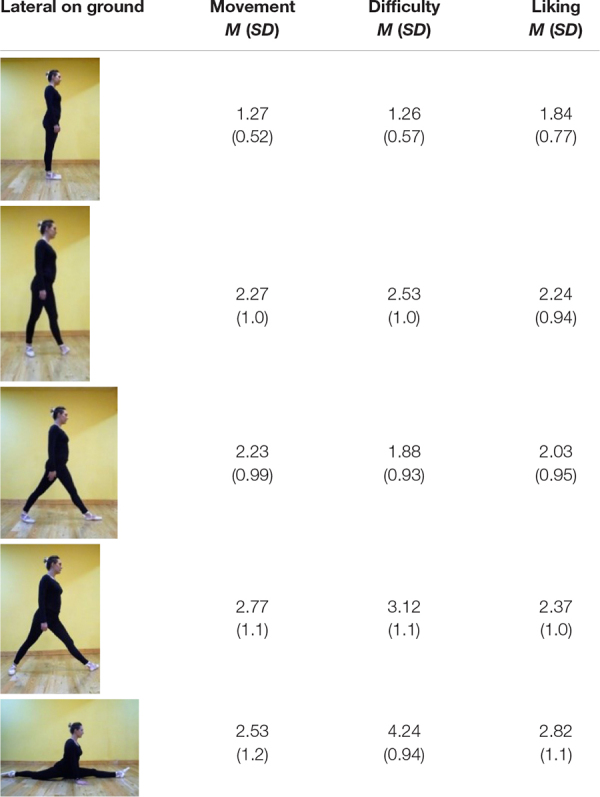

**FIGURE 3:**
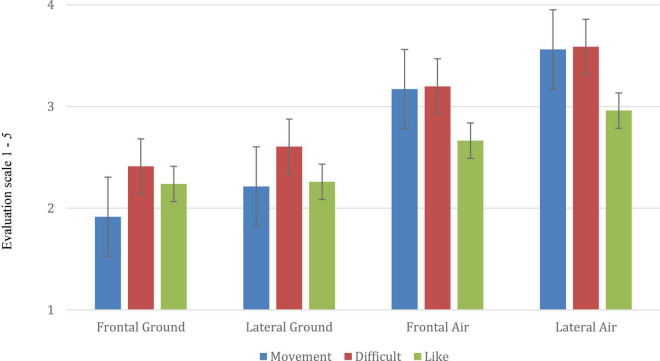
Mean scores for the three variables, *Movement*, *Difficulty* and *Liking* for the four poses, F*rontal On-ground*, *Lateral On-ground*, *Frontal In-air* and *Lateral in-air*.

To summarize the findings, mean scores of the three dependent variables (*Movement*, *Difficulty* and *Liking* per view), *On-ground* and *In-air*, are in Figure 3.

We will offer descriptive statistics and then proceed to reliability analyses. For ease of comprehension, we comment briefly on some findings. *Movement* and *Difficulty* were influenced greatly by leg angle, and *Liking* less, and *In-air* scores were higher than *On-ground, w*ith *Lateral In-air* scores especially high. We will note a restriction of range for *Liking*, and that symmetry may matter.

Overall, ranges were considerable and, as Figure 3 suggests, floor and ceiling effects were avoided. For *Movement*, the range of mean scores per pose was 2.75, from 1.18 (*Frontal, on ground*, 0°) to 3.93 (*Lateral, in-air*, 180°). For *Difficulty* the range of scores was 3.59, from 1.15 (*Frontal, on ground*, 0°) to 4.74 (*Lateral, in-air*, 180°). For *Liking*, the range was only 1.68, from 1.84 (*Lateral on ground*, 0°), to 3.52 (*Lateral, in-air*, 180°). For individual poses, the *Difficulty* range was greater than that for *Movement* and double that for *Likin*g.

**FIGURE 4:**
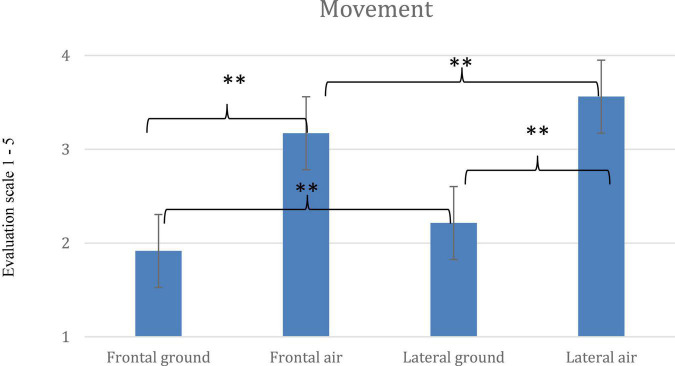
Mean scores for *Movement* and significant interaction for the four poses, *Frontal On-ground*, *Lateral On-ground*, *Frontal In-air* and *Lateral In-air* (error bars indicate standard errors of the means; ^**^ = < 0.001).

**FIGURE 5:**
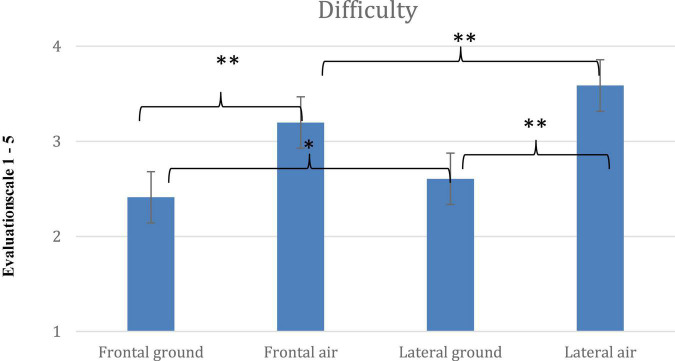
Mean scores for *Difficulty* and significant interaction for the four poses, *Frontal On-ground*, *Lateral On-ground*, *Frontal In-air* and *Lateral In-air* (error bars indicate standard errors of the means; * = < 0.05; ^**^ = < 0.001).

**FIGURE 6:**
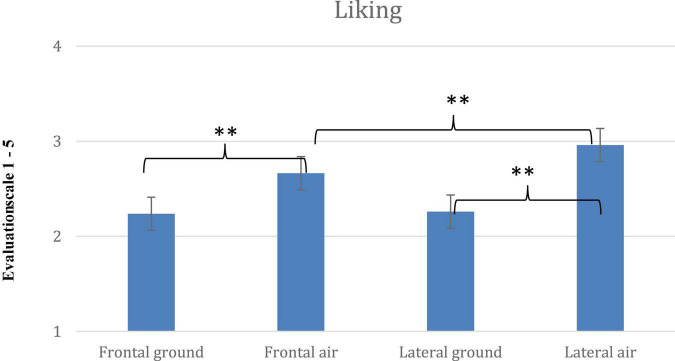
Mean scores for *Liking* and significant interaction for the four poses, *Frontal On- ground*, *Lateral On-ground*, *Frontal In-air* and *Lateral In-air* (error bars indicate standard errors of the means; ^**^ = < 0.001).

Comparisons of means of the dependent variables (*Movement*, *Like* and *Difficulty*) for each of the 4 pose conditions are shown in Figures 4–6. Across the dependent variables, *In-air* conditions had especially high scores.

Repeated-measure ANOVAs were conducted for each of the dependent variables, *Movement*, *Difficulty* and *Liking*, with a 5 × 2 × 2 factorial design: *Leg angles* (5 levels: 5 degrees of spread) × *Vantage-point* (2 levels: frontal and lateral) × *Elevation* (2 levels: on-ground, in-air).

### Movement

For *Movement*, the main effect of *leg angles* was significant [*F*(4, 396) = 84.884; *p* < 0.001, η^2^ = 0.462]. Greater angles meant more movement. The main effect of the camera *Vantage-point* was significant [*F*(1, 99) = 74.400; *p* < 0.001, η^2^ = 0.429]. Lateral-view movement ratings were higher than frontal. Similarly, the main effect of air or ground *Elevation* was significant [*F*(1, 99) = 151.222; *p* < 0.001, η^2^ = 0.604]. *In-air* ratings were much higher than *On-ground.*

For *Movement*, a three-way interaction (*Leg angles* × *Vantage-point*× *Elevation*) was significant [*F*(4, 396) = 5.798; *p* < 0.001, η^2^ = 0.055]. *Post-hoc t*-tests were run. Ratings for *In-air* and *Lateral* poses were notably high—reaching *M* = 3.93 for the splits.

Comparisons using the two most extreme poses—the splits and 0°—are clear. *Lateral In-air* 0° (*M* = 3.35) offered more movement than *Frontal In-air* 0° (*M* = 2.93) *p* < 0.001; *d* = 0.29, which offered more than *Lateral On-ground* 0° (*M* = 1.27; *p* < 0.001; *d* = 1.94); *Lateral On-ground* was not different from *Frontal On-ground* 0° (1.18), NS.

Regarding the 4 splits poses with legs 180° apart: *Lateral In-air* (*M* = 3.93) was rated higher than *Frontal In-air* (*M* = 3.33, *p* < 0.001; *d* = 0.58), which was higher than *Lateral On-ground* (*M* = 2.53), *p* < 0.001; *d* = 1.07), which was similar to *Frontal On-ground* (*M* = 2.34) = NS.

*Lateral In-air* poses are literally part of upward motion and may also benefit from implying extra movement, to the side. *Movement* scores increase steadily with leg angle in the *Lateral* positions *in-air*—a leap forward as well as upward—and less steadily in other conditions.

### Difficulty

The main effect of leg-angle in the ANOVA for *Difficulty* (*Leg angles* x *Vantage-point* x *Elevation*) was significant [*F*(4, 396) = 411.613; *p* < 0.001, η^2^ = 0.806], with larger angles rated more difficult. The main effect of *Vantage-point* was significant [*F*(4, 396) = 58.321; *p* < 0.001, η^2^ = 0.371], with poses shown in the *Lateral* viewpoint rated more difficult. The main effect of *Elevation* was also significant [*F*(1, 99) = 111.904; *p* < 0.001, η^2^ = 0.531]. *In-air* difficulty ratings were higher than *On-ground.*

The three-way interaction (*Leg angles* × *Vantage-point*× *Elevation*) was also significant [*F*(4, 396) = 11.008; *p* < 0.001, η^2^ = 0.100]. *Post-hoc t*-tests were run. Again, comparisons within the two most extreme poses –0° and the splits—are informative.

For 0°: *Lateral In-air* (*M* = 3.27) was rated more difficult than *Frontal In-air* (*M* = 2.8), *p* < 0.001; *d* = 0.4) and both were more difficult than *Lateral On-ground* (*M* = 1.26) and *Frontal On-ground* (*M* = 1.15), which were alike (and with ground support, standing *Difficulty* should indeed be minimal, and camera vantage point should be immaterial).

Regarding the splits: *Frontal In-air* (4.74), *Frontal On-ground* (4.42) and *Lateral On-ground* (4.24) were alike, and each was more difficult than *Lateral In-air* (3.27) (all *p* < 0.001). The splits is difficult, but *Lateral In-air* (3.27) may seem like a leap upwards and forward, and thereby close to part of an observer’s skill set.

*Difficulty* of *Frontal On-ground* poses, including straddling poses that are hard to maintain, increased more steadily with leg angle (in order, from 1.15 to 1.75, 1.99, 2.75, and 4.42) than poses in other conditions, but the pose rated most difficult was the lateral in-air splits (4.74) that has no ground support.

### Liking

In general, the larger the angle the more the pose was liked, but no condition offered consistent increases in *Liking*, which can be expected given the small range of *Liking* scores, and high scores given to 0° in-air poses. The ANOVA for *Liking* included *Leg angle* x *Vantage-point* x *Elevation.* The main effect of leg angle was significant [*F*(4, 396) = 102.956; *p* < 0.001, η^2^ = 0.510]. The larger the angle the higher the rating. The main effect of *Vantage-point* was significant [*F*(1, 99) = 18.293; *p* < 0.001, η^2^ = 0.156]. *Lateral* poses were liked slightly more than *Frontal* ones The main effect of *Elevation* was also significant [*F*(1, 99) = 53.265; *p* < 0.001, η^2^ = 0.350], with in-air poses rated higher.

The three-way interaction (*Leg angle* × *Vantage-point* × *Elevation*) was also significant [*F*(4, 396) = 5.738; *p* < 0.001, η^2^ = 0.055]. *Post-hoc t*-tests were run. Once again, comparisons within the two most extreme poses—0° and the splits—are informative.

For 0° poses, the *Lateral In-air* pose (*M* = 2.76) was liked more than any other (*p* < 0.001), and the *Frontal In-air* pose was preferred to the *On-ground* poses which were alike.

Regarding the splits: the *Lateral In-air* pose (*M* = 3.52) was liked above the others, including *Frontal In-air* (*M* = 3.29; *p* < 0.05; *d* = 0.17), and *p* < 0.001 for the other comparisons. *Frontal In-air* was preferred to the on-ground poses (*p* < 0.05), and *Frontal On-ground* (*M* = 3.06) was liked more than *Lateral on-ground* (*M* = 2.82) (*p* < 0.05; *d* = 0.11). Along with notable difficulty, *Lateral in-air* has upward and sideways movement options. The *Lateral On-ground* pose is less symmetrical than the frontal poses.

*Liking* scores increased slightly more steadily with leg angle for *Frontal on-ground* poses than for other poses, with the *Liking* scores decreasing with leg-angle only once, and slightly, from 1.91 for standing to 1.80 for legs-slightly apart (their symmetry may matter). For *Lateral in-air* poses, *Liking* scores begin with high ratings for 0° (restricting range) and eventually rose slightly with leg-angle—in order, 2.76, 2.76, 2.73, and then up to 3.03 and finally 3.52. *Lateral on-ground Liking* ratings (a small range) decreased once with leg-angle, and *Frontal in-air Liking* ratings (with high ratings for 0° poses restricting the range) decreased twice with leg angle.

Results of Pearson correlation indicated that *Movement* correlated positively with both *Difficulty* (*r* = 0.747; *p* < 0.001) and *Liking* (*r* = 0.808; *p* < 0.001). However, *Difficulty* correlated positively and very impressively with *Liking* (*r* = 0.949; *p* < 0.001), close to ceiling. To consider the relationship between the three variables, *Liking*, *Movement* and *Difficulty*, a multiple regression analysis was conducted. The regression model with the two predictors, *Difficulty* and *Movement*, explained 92% of the variance [*R*^2^ = 0.92; *F*(2, 17) = 102.3, *p* < 0.001]. *Difficulty* significantly predicted *Liking* (β _diff_. = 0.37, *p* < 0.001); *Movement* also predicted liking but with a weaker effect (β _mov_ = 0.14, *p* < 0.05), indicating *Difficulty* has an extra effect over and above *Movement*.

## Discussion

For observations of images of motion, the relations between indications of movement, apparent difficulty and personal liking have been debated (Arnheim, 1974; Topolinski, 2010; Cross et al., 2011; Orlandi et al., 2020a,b). Here we consider these factors in reports of impressions of a dancer in formal poses showing a range of leg angles.

In the present case, as hypothesized, *Movement* ratings increase with limb angle, especially *in-air* in *lateral* views. In all 4 conditions—*Frontal* and *Lateral In-air*, and *On-ground*—the most-spread pose had higher *Movement* ratings than the least-spread. On-ground poses imply motion indirectly (like the *Lightning-Bolt* posture), and in-air images literally freeze mid-flight motion. Studies using verbal reports and cortical responses show that static images can convey implicit motion (Kourtzi and Kanwisher, 2000; Urgesi et al., 2006; Proverbio et al., 2009; Orgs et al., 2013). Here, leg extension in static pictures of a dancer conveyed motion. Likewise, for pictures of horses in gaits such as walk and trot, the more the legs were extended fore-and-aft the more motion observers reported (Mastandrea and Kennedy, 2018).

There may have been some influences from symmetry on *Liking*, but the clearest effect to consider is that *Liking* correlated less with *Movement* than with *Difficulty*. In this vein it is worth noting that Topolinski (2010) reported that liking increased with training in actual muscle movements, and muscle training is related to skill. Topolinski (2010) was concerned with visual muscles, however, and the present study concerns observation of large-muscle activity.

As expected, *Movement* ratings were highest for *In-air* conditions. Their ratings increased steadily with spread in the *lateral* views, which likely suggest a jump forward as well as a jump up, attracting the highest movement scores and the greatest movement range. In contrast, *Frontal In-air* views had very little range, though it should be said that, in some support of the movement-angle hypothesis, the lowest score was for 0° and the maximum scores were for 65^°^ and 180° spreads. More emphatically and convincingly, at every angle, 0^°^–180°, the *lateral* views are rated higher than the *frontal* views for motion. The *frontal* view may merely suggest a jump upwards, and *lateral* views suggest upwards and in the direction to the side she faces.

*On-ground Movement* scores, which at best imply movement, were low and ranged from close to baseline, for standing erect, to midrange for the splits. Increases in *on-ground* ratings may be due to implied motions, such as deviating from standing, a default pose. The enhancement could also be due to increased effort required to maintain a straddling pose as limbs spread, since for both *frontal* and *lateral on-ground* views, the maximum *Movement* scores on the ground were biomechanically inefficient poses, legs spread 65°, and short of the highly difficult splits. At every angle, *Lateral On-ground* views are rated higher than *frontal* images, supporting the interpretation that *frontal* views suggest holding the pose and *lateral* views may indicate the end of the act of stepping forward.

As noted in regression analysis, *Movement* scores correlated positively with *Difficulty* and *Liking*, but the highest correlation, essentially at ceiling, was between *Difficulty* and *Liking* (*r* = 0.949). The explanation may be that dance entails extensively practiced skilled movements. It takes some skill to adopt and hold on-ground poses, and even the standing dancer in the present study is in a formal pose, head high, shoulders back, elbows by the side, torso erect. It may be skill in adopting a trained pose attracts *Liking* scores. Since all the poses are formal, *Liking* ratings have a restricted range. But some of the poses are especially difficult to master, particularly leaps with full-extension leg spread extending fore-and-aft of the dancer or to the dancer’s left and right. *Difficulty* may be a proxy for skill, particularly a skill level beyond the observer’s. If so, rather than motion being liked, or difficulty being liked, it is skill that is liked. In a study on timing of actions, dance movement sequences judged more effortful, and more difficult (*reproducible* by the observer), were more aesthetically pleasing (Orlandi et al., 2020a). Besides *Difficulty*, in the present study with static pictures of a dancer, increase in *Movement* is related to *Liking*.

We should note that the dancer’s leg positions are accompanied by relatively fixed arms. This is a standard feature of some well-known dances. In Celtic dances, the arms are downwards in Irish step dance, and upwards, above the shoulders, curled, in Scottish highland dance, as in classical ballet’s 5th position of the arms, en haut in the Cecchetti method. In future research, combinations of arm and leg angles could test the limb-angle/movement hypothesis. Thereby, the *Liking* range might increase, as the downward arm postures here offer a subdued range of expressions (Ross and Flack, 2019).

In photographs of an actual dancer, as here, as a matter of practicality, some features of the feet, arms, torso and head are not as controlled as they would be in drawings. For example, in Figure 1’s lateral images, the dancer’s head is erect in the 0° pose, but it comes forward steadily in the 20–180° images. In a future study this would deserve attention.

The results here may be important and relevant for the psychology of art and motion depiction. A photograph of a dancer, legs extended, far apart, can convey a vivid sense of motion, offer a difficult pose and yet be highly liked. Designers and artists may find it useful to know that large angles between limbs boost implied motion, and just as helpful to know that postures difficult to achieve may be highly admired. Perception psychologists may find that high levels of implied motion in pictures of real-life dancers have many cortical and perceptual effects e.g., motion after-effects (Winawer et al., 2008).

## Conclusion

In conclusion, in ratings of photographs of a dancer in-air (especially) and on-ground (slightly), in frontal and lateral view, increase in leg angle suggested more motion. Evidently, limb extension is a factor suggesting motion in static pictures. Also, in-air poses are in the midst of an actual leap, and on-ground poses may suggest motion in indirect fashion. *Lateral* views may benefit from suggesting movement sideways. *Difficulty* and *Liking* scores increased from erect poses to the splits. However, *Liking* (small range) was more related to *Difficulty* (large range), and possibly skill, than *Movement* (intermediate range).

## Data Availability Statement

The raw data supporting the conclusions of this article will be made available by the authors, without undue reservation.

## Ethics Statement

Ethical review and approval was not required for the study on human participants in accordance with the local legislation and institutional requirements. The patients/participants provided their written informed consent to participate in this study. Written informed consent was obtained from the individual(s) for the publication of any potentially identifiable images or data included in this article.

## Author Contributions

SM conceived the idea of this study, and performed the testing, data collection, and statistical analysis. SM and JK interpreted the results, wrote, and approved the final version of the manuscript. Both authors contributed to the article and approved the submitted version.

## Conflict of Interest

The authors declare that the research was conducted in the absence of any commercial or financial relationships that could be construed as a potential conflict of interest.

## Publisher’s Note

All claims expressed in this article are solely those of the authors and do not necessarily represent those of their affiliated organizations, or those of the publisher, the editors and the reviewers. Any product that may be evaluated in this article, or claim that may be made by its manufacturer, is not guaranteed or endorsed by the publisher.
